# “Building bridges”—communication education for residents in radiology: a scoping review

**DOI:** 10.1186/s12909-024-05660-3

**Published:** 2024-06-14

**Authors:** Ruiting Zhang, Xiaopei Xu, Xiao Luo, Peiyu Huang

**Affiliations:** Department of Radiology, The Second Affiliated Hospital of Zhejiang University, School of Medicine, 88# Jiefang Road, Hangzhou, China

**Keywords:** Communication, Education, Radiology residents, Scoping review

## Abstract

**Background:**

Good communication is an important professional attribute for radiologists. However, explorations of communication education and their outcomes in radiology residents are sparse. This scoping review aims to evaluate the existing literature on communication education for radiology residents, identify gaps in current practices, and suggest directions for future studies.

**Methods:**

A scoping review following the six-step approach of Arksey and O’Malley was undertaken. We searched through PubMed, Embase, ERIC, and Web of Science databases, focusing on communication education in radiology residents.

**Results:**

Sixteen of the 3096 identified articles were included in the analysis. Most studies (13/16) originated from the United States. The studies varied in study design, including quantitative, qualitative and mixed-methods approaches. The sample sizes of most studies were small to moderate, with more than half of the studies had fewer than 30 participants. The identified studies predominantly focused on communication with patients and healthcare professionals. The need for communication education, the efficacy of specific communication education programs, and the capability of some assessment tools for evaluating residents’ communication skills were investigated.

**Conclusions:**

This scoping review reveals the gap between the need for communication education and the lack of comprehensive education programs in radiology residents globally. Future studies should develop tailored interventions and use reliable assessment tools, engaging more participants with extended follow-up periods, and expand the scope of communication training to include all relevant stakeholders.

**Supplementary Information:**

The online version contains supplementary material available at 10.1186/s12909-024-05660-3.

## Introduction

Communication refers to the process of transmitting information from one person or group to another through various means [1]. Traditionally, radiologists interacted with clinical physicians primarily through diagnostic reports, facilitating an indirect form of communication. However, contemporary practices have seen a shift towards more direct engagement, and the interactions encompass not only clinicians and colleagues but extending to patients and their families as well [2, 3]. The theme of the 2022 European Congress of Radiology (ECR), “building bridges” emphasized these changes, underscoring the necessity for radiologists to enhance their communication skills, so as to foster effective interactions with patients, clinical partners, scientists, and the industry [4].

As we recognize the rising importance of communication in radiology practices, it is crucial to examine the present situation of communication education in radiology residency training. Since 1999, the Accreditation Council for Graduate Medical Education (ACGME) has identified communication as one of six core competencies required for all residency trainees [5, 6]. Into the 21st century, many radiologic professional organizations, including the American College of Radiology (ACR), The American Board of Radiology (ABR), and the European Society of Radiology (ESR), have initiated programs and provided guidelines for communication education and evaluation [7–9]. These efforts aim to help radiology residents understand the significance of communication, enhance their communication skills, and finally improve their competencies.

However, despite these efforts, there is a noticeable lack in the literature concerning the characterization and evaluation of communication education within radiology residencies. There is a pressing need for a comprehensive synthesis of existing literature to scope the targets, interventions, and outcomes of these programs. We aimed to evaluate the existing literature on communication education for radiology residents, address the current gaps in our understanding, and suggest directions for future studies.

## Methods

Given the inherent qualitative nature of the literature and the anticipated heterogeneity of initiatives, a scoping review was deemed most appropriate for the synthesis of the relevant literature. This scoping review applies the approaches proposed by Arksey and O’Malley and modified by Levac and Colquhoun [10, 11], which consist of six stages: (1) identify the research question, (2) identify relevant studies, (3) select the studies, (4) chart the data, (5) summarize and report the results, and (6) consult with stakeholders. The Preferred Reporting Items for Systematic Reviews and Meta-Analysis for Scoping Reviews (PRISMA-ScR) criteria guided the reporting of the review [12].

### Identifying the research question

We aimed to answer the following research questions: (1) What is known about communication education in radiology residency training? (2) What topics are awaiting further investigation?

### Identifying relevant studies

Pubmed, Embase, Education Resources Information Center (ERIC) and Web of science databases were searched for all English-language studies from 1950 to the date on which the search was performed (October 10th, 2023). A search string was built and tailored to each database, searching for terms in titles, abstracts, keywords, and MeSH terms. The full search strategy for each database was detailed in the Appendix.

### Study selection

Identified records were imported into EndNote (version X5, Clarivate Analytics, Philadelphia, United States), and duplicates were removed. Two independent reviewers (RZ and XX) screened titles and abstracts for eligibility. Relevant reports were retrieved and assessed in full text against the inclusion criteria. Full-text reports that did not meet the inclusion criteria were excluded, and the reasons for exclusion were registered. Disagreements between the two reviewers were resolved through discussions with a third reviewer (XL). The reference lists of the initially retained reports were hand searched. The selection procedure for the reference reports was the same as described above.

### Charting the data

We first developed an initial data abstraction form by including descriptive contents such as study characteristics, topic areas, and methodologies. A detailed revision was then performed by reading through a random selection of five articles, and the procedure was modified iteratively for final presentation of the data. The included studies were divided equally between the two authors (RZ and XX), uncertainties about abstraction were marked and later reviewed again.

### Consulting with stakeholders

According to Arksey and O’Malley’s sixth stage, we presented our review and findings with four stakeholders, two of whom were radiology residents, one of whom was an experienced attending radiologist, and the other was an engineer and responsible for the research projects in our department. The stakeholders read through the entire manuscript, provided written feedback on the presentation of the main findings, and suggested relevant issues for discussion. The comments were included in the authors’ deliberations of the presentation of the results and in the discussion of the results.

## Results

**Fig. 1:**
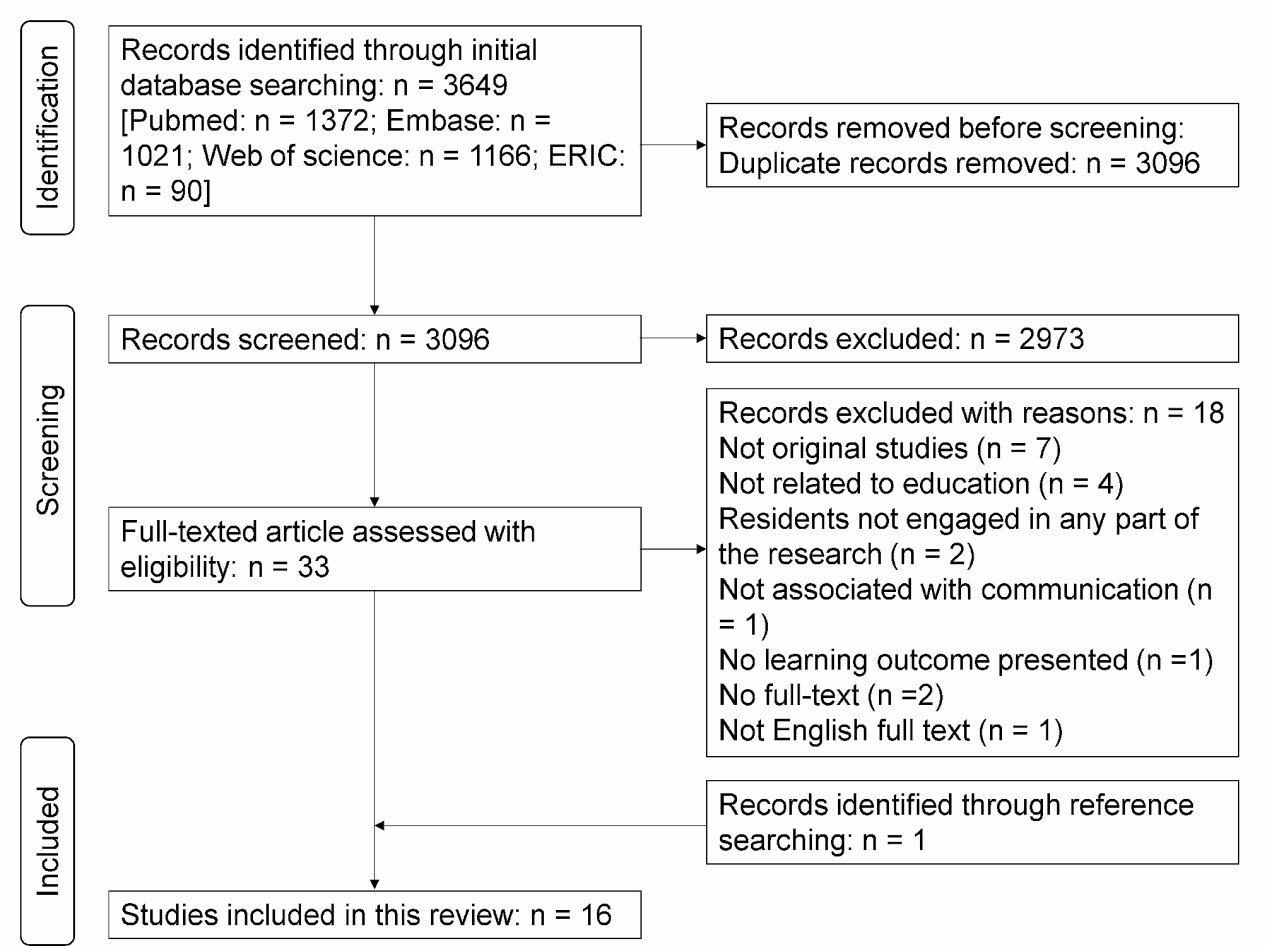
PRISMA flow diagram for study selection

We initially retrieved 3649 citations from the Pubmed, Embase, ERIC and Web of science databases. After removing duplicates, a total of 3096 citations remained. We then excluded 2973 citations after screening the titles and abstracts on the basis of the inclusion and exclusion criteria shown in Table 1. The subsequent screening of the remaining 33 full-text articles led to the exclusion of 18 articles. Reasons for exclusion included: not being original research, irrelevance to education or communication, lack of resident involvement, absence of reported outcomes, or availability in English abstracts only. Through reference searching, we identified an additional article. Finally, 16 articles satisfying the inclusion criteria were retained for data analysis. Our screening procedure is summarized in Fig. 1.

**Table 1:** Inclusion and exclusion criteria

Criterion	Inclusion	Exclusion
Study focus	Communication—including communication with patients, colleagues in the radiology department, physicians, health providers, peers, and etc. Every process of education—including needs adjustment, planning, implementing interventions, and outcome assessment.	/
Population	Radiology residents	Medical students, fellows, residents from other departments
Type of articles	Original, peer-reviewed research	Reviews, point/opinion
Language	English	Non-English

### Study characteristics

Most of the articles came from the United States (*n* = 13); the rest were from Spain (*n* = 1), China (*n* = 1) and Pakistan (*n* = 1).

The articles varied in study design: Ten were quantitative studies, three were qualitative studies, and three were mixed-method studies.

The sample sizes in most studies were small to moderate, with over half of the studies included fewer than 30 participants. However, a notable exception was a study from China, which had a sample size of 1003 participants [13].

### Summary of target communicatee

Table 2 provides a summary of the target communicatees. Seven studies centered on communication with patients and their families, while five studies focused on interactions with health professionals, including physicians and radiologists. Four studies did not specify their target communicatee, and emphasized on the development of general communication skills.

**Table 2:** Study characteristics and target communicatee

Reference	Country	Study type	Target communicatee
Lafleur et al. [18]	US	Mixed methods	General communication, mainly with radiologists
Narayan et al. [14]	US	Quantitative	Patient communication
Fairchild et al. [22]	US	Quantitative	Patient communication
DeBenedectis et al. [23]	US	Qualitive	General communication, mainly with patients
Brown et al. [16]	US	Quantitative	Patient communication
Salama et al. [24]	US	Quantitative	Between radiologists and physicians
Goldman-Yassen et al. [20]	US	Quantitative	Between radiologists and physicians
Nadeem et al. [17]	Pakistan	Quantitative	General communication, mainly with patients
Pino-Postigo et al. [21]	Spain	Quantitative	Communication with peers
Lang et al. [25]	US	Qualitive	General communication
Lown et al. [19]	US	Mixed methods	Patient communication
Ding et al. [13]	China	Quantitative	General communication
Whittington et al. [15]	US	Qualitive	Interdisciplinary communication
Brown et al. [26]	US	Quantitative	General communication, mainly with patients
Majid et al. [27]	US	Mixed methods	General communication
Itri et al. [28]	US	Quantitative	General communication

*US* United States

### Summary of the study topics, methods and main findings

To systematically organize and analyze the collected data, we grouped the identified studies into three main topics: Topic (1) Investigating the need for communication education among radiology residents; Topic (2) Developing assessment tools for evaluating radiology residents’ communication skills; Topic (3) Evaluating the effectiveness of specific communication education programs. Of the 16 studies analyzed, two focused on Topic 1, ten focused on Topic 2 and five focused on Topic 3, including one study explored both Topic 2 and 3 (Table 3).

**Table 3:** Summary of the study topics, methods and main findings

Topic	Reference	Methods/Sample/Intervention/Outcome measurements	Main findings
***Topic 1.*** *Investigating the need for communication education among radiology residents*	Narayan et al. [14]	• Survey and descriptive statistics • 73 radiology residents	A large majority (91.8%) of radiology residents have communicated test results to patients, yet few have received training in how to communicate these results. 79.4% agreed or strongly agreed that additional training in conveying results to patients would be beneficial.
	Whittington et al. [15]	• Survey and descriptive results • 28 radiology residents	Radiology students in clinical rotation were able to identify instances of failed inter-disciplinary communication within the clinical setting.
***Topic 2.*** *Developing assessment tools for evaluating radiology residents’ communication skills*	Lafleur et al. [18]	• Participants: 15 radiologist-resident pairs and 15 internist-resident pairs • Method for evaluation: supervisors’ verbal feedback • Descriptive statistics	Case discussions represent frequent opportunities for substantial feedback on intrinsic roles, largely aligned with the clinical case. Supervisors predominantly offered monologues of advice and agreements.
	Brown et al. [16]	• Participants: 5 radiologists and 4 non-radiologists • Method for evaluation: Kalamazoo Communication Skills Assessment instrument • Reliability	The adapted communication skills assessment instrument is highly relevant for radiology, having moderate interrater reliability.
	Nadeem et al. [17]	• 42 radiology residents • Method for evaluation: a binary checklist and a Likert type rating scale • Consistency and reliability	Both checklists and rating scales can serve as satisfactory assessment tools for communication and interpersonal skills using objective structured and clinical examination with the assistance of faculty and standardized patients.
	Ding et al. [13]	• 1003 radiology residents • Method for evaluation: simulation video and standardized patient conversation evaluation model • Discrimination ratio and descriptive statistics	The simulation video demonstrated a stable and better acceptable construct for assessing radiology residents’ communication skills.
***Topic 3.*** *Evaluating the effectiveness of specific communication education programs*	Fairchild et al. [22]	• 21 radiology residents • Intervention: Reading material, lecture, Role-play activities • Pre- and post-intervention evaluation by attending radiologist and standardized patient (SP) using scales	Checklist-based consent training improved radiology residents’ ability to obtain informed consent.
	DeBenedectis et al. [23]	• 8 radiology residents • Intervention: debriefing sessions • Pre- and post-intervention evaluation by patient-actor, faculty and residents themselves using scales	Practicing their communication skills boosted their ability to communicate. The educational feedback from the training sessions was useful, which contradicts their self-evaluations.
	Salama et al. [24]	• 20 radiology residents and 150 internal medicine physicians and medical students • Observational, clinical imaging rounds (CIR) • Surveys after the CIR completed by radiology residents and internal medicine participants	Resident-driven imaging rounds provide a valuable opportunity to improve communication, education, and patient care.
	Goldman-Yassen et al. [20]	• 161 medicine residents • Observational, weekly radiology rounds • Surveys completed by participating medicine residents	Radiology resident-led educational medicine rounds promote cross-specialty collaboration, further educate trainees, and directly affect patient management.
	Pino-Postigo et al. [21]	• 28 radiology residents • Intervention: clinical session meeting in a virtual place • Self-evaluation after the intervention by completing questionnaires	Second Life can be used effectively to train oral communication skills in public, in an environment perceived as attractive and suitable for learning.
	Lang et al. [25]	• 28 trainees (radiology residents and fellows) • Intervention: preassessment survey, initial videotaped role-play, self-study of an electronic Web-based teaching module, a debriefing session and the microteaching exercise • Descriptive results	The microteaching exercise is suitable to such pursuit and permits the level of reflection sought by educators and the ACGME as a prerequisite for refinement of skills.
	Brown et al. [26]	• 109 radiology residents • Intervention: workshop including three modules (bad news, medical errors and radiation risks) • Pre- and post-intervention self-evaluation	After participation, fewer trainees reported unwillingness to disclose the error despite medicolegal concerns. Most respondents after participation desired additional communication training on error disclosure, general communication, and radiation risks.
	Majid et al. [27]	• 5 radiology residents • Intervention: 6 simulated scenarios and debriefing sessions • Post-intervention (immediate and 4-year follow-up) evaluation by faculty and staff	Simulation-based communication skills training is effective and long lasting.
	Itri et al. [28]	• 21 radiology residents • 1-day experiential communication skills workshop with Myers-Briggs Type Indicator and conflict management sessions including group discussion, role-play, and simulation • Post-intervention self-evaluation with questionnaires	The workshop, designed to improve radiology trainee competency and comfort with communication and conflict management, is an effective educational initiative to teach important skills to radiology trainees that are not learned well using traditional didactic approaches.
Both ***Topic 2*** and ***3***	Lown et al. [19]	• 9 radiology residents • Method for evaluation: Radiology Communication Skills Assessment Tool (RCSAT) • Intervention: “patients as teachers” session, a radiology objective structured clinical examination, and communication skills curriculum • Pre- and post-intervention evaluation by patient-teacher and participants themselves	Educational curricula on communication about difficult information can be implemented in radiology training programs. Radiology residents’ performance can be assessed using a communication skills assessment tool during standardized patient-teacher encounters.

Both the two studies focusing on Topic 1 utilized surveys and presented their findings in a descriptive manner. They both identified needs for communication education from radiology residents [14, 15].

For Topic 2, two studies developed and validated their assessment tools [16, 17], while the other three introduced evaluation methods and described their approaches as effective and useful [13, 18, 19].

For Topic 3, two studies employed observational methods [20, 21], while the others utilized various interventions, such as lectures, role-playing activities, and simulated scenarios [19, 22–28]. Across all the nine studies, the findings were uniformly positive, demonstrating that the applied interventions enhanced the participants’ communication skills, facilitated collaboration, and impacted patient management positively.

## Discussion

In this scoping review, we retrieved data from four major databases and identified 16 studies focusing on communication education for radiology residents. The included studies varied in methodologies including quantitative, qualitative, and mixed-method approaches. Most studies (13/16) originated from the United States. The target communicatees were patients and health professionals. The need for communication education, the capability of some assessment tools for evaluating residents’ communication skills, and the efficacy of specific communication education programs were investigated.

Notably, the majority of related studies originate from the United States. Four potential explanations exist for this phenomenon. First, the six core competencies, including communication skills, were proposed by the ACGME in the United States [5]. The earlier recognition of the importance of communication could have led to an earlier onset of related studies in the United States. Second, radiology is a specialty that heavily relies on advanced technology and equipment, and residency education in this field typically occurs in more developed centers. For example, our research did not find any studies from Africa, which may be due to the nascent stage of radiology education in the region, where communication training has not yet been prioritized. Third, the level of attention to different topics varies by region. For instance, a significant study involving 1003 radiology residents was conducted in China aimed at developing a tool to assess residents’ communication skills [13]. In their discussion, the researchers expressed intentions to use this tool for further research into communication education, indicating that a continued focus in this area might reveal more studies from diverse regions in the future. Lastly, it is important to mention that our research was conducted using English-language searches, which may have missed studies from non-English-speaking countries, such as those in Continental Europe. This represents a limitation of our study. We believe that the geographical imbalances identified in the current research also point to directions for future studies. There is substantial potential for further research in regions outside of the United States.

Compared to the literatures of other medical disciplines, communication education studies specifically for radiology residents are relatively sparse. For instance, the field of surgery has produced 33 relevant articles according to a scoping review conducted up until April 2020 [29], while internal medicine has contributed 32 articles by 2018 [30]. The phenomenon might be partly explained by the inherent nature of radiology work that encounters less direct patient interaction. However, according to one of the two studies addressing the need for communication education, 91.8% of residents reported that they communicated results with patients, while only 16.4% received relevant training, and 79.4% agreed or strongly agreed that additional training would be helpful [14]. Furthermore, our observations from daily work and communications with stakeholder suggest a genuine need for enhanced communication skills within the radiology residents. There is a necessity for detailed studies that explore the communication needs of radiology residents, detailing what skills they lack and what they aim to gain through education, combined with the expectations of those they communicate with, such as patients, physicians from other departments, technicians, and scientific researchers.

We identified five studies aimed at developing assessment tools for evaluating the communication skills of radiology residents, and two of them validated the reliability of their instrument. However, none of the five studies had validated the reliability, accuracy, discrimination ratio and time-effectiveness systematically. The development of such assessment tools is indeed challenging. The diversity in communication scenarios within the scope of radiology work requires different evaluation methods. Breaking bad news to patients and expressing disagreement with colleagues require different communication skills and thereby need to be assessed with specific approaches [8]. In addition, the subjective nature of communication skills evaluation can introduce variability. Future studies could aim to refine existing assessment tools, ensuring their validity and reliability across diverse settings and scenarios, and explore innovative assessment methods, such as simulation-based evaluations.

Notably, all the ten studies focusing on specific communication education programs presented positive outcomes, demonstrating the effectiveness of the evaluated programs on improving the residents’ communication skills. However, the studies also shared some common limitations: The sample size were small to moderate (smallest: *n* = 5, largest: *n* = 161) [20, 27]; The self-rating of competence and comfort adopted as outcome does not necessarily translate into improved competency in communication; Long-term sustainability of the acquired communication skills after intervention were not investigated except in one study [27]. These limitations reduce the credibility and preclude the generalizability of their findings. Future studies with large sample size, objective outcome measurements and long-term follow-up would be beneficial.

Moreover, our categorization of the three topics is based on a summary of the 16 articles we included, which reflects the current state of research and the topics covered, and not necessarily encompasses all potential content within the broad research field of communication education. This categorization may carry the risk of oversimplifying existing research and introduces some degree of authorial subjective bias. Our scoping review primarily serves to provide an overview; detailed specifics should be sought within the respective references.

In future, radiologist would embrace closer relationship with not only patients and other healthcare professionals, but also engineers, industry partners, and researchers. Effective communication could “build bridges” between radiologists, science and technology. However, we found no literature in this field currently, which should be a focal point for future research.

This scoping review has limitations. First, we only included studies in peer-reviewed journals and not gray literature. This restriction may affect the whole picture of mapping the communication education in radiology residents. Second, only English publications were included. The excluded publications could neglect the potential influences of studies from non-English speaking countries, and would affect the final result of the regional distribution of the included studies.

In summary, this scoping review reveals the gap between the recognized necessity for communication education and the lack of comprehensive education studies in radiology residents around the world. Future studies should develop tailored interventions and use reliable assessment tools, engaging more participants with extended follow-up periods, and expand the scope of communication training to include all relevant stakeholders. We believe that communication education is crucial for harnessing the multidisciplinary nature of radiology and building bridges for future radiologists aiming to improve patient care and foster innovation within the field.

### Electronic supplementary material

Below is the link to the electronic supplementary material.

Supplementary Material 1

## Data Availability

The datasets supporting the conclusions of this article are included within the article and its additional file.
