# Supplementary material for: “Building bridges”—communication education for residents in radiology: a scoping review
Source: BMC Med Educ. 2024 Jun 14;24:662. doi: 10.1186/s12909-024-05660-3 (PMC11179299; doi:10.1186/s12909-024-05660-3)
Supplement: Supplementary file 1 — Supplementary Material 1 [file 12909_2024_5660_MOESM1_ESM.docx]

**Appendix**

**Search strategy used in the databases**

**Pubmed**

1950 to 2023 October 10^th^

1. radiology or ‘‘medical imaging’’ or ‘‘diagnostic imaging’’ or ‘‘diagnostic radiology’’, Ti and Ab: 96,330 results.
2. resident* or residenc* or "clinical attachment*" or "clinical rotation*" or fellowship or internship or physician* or trainee* or postgraduate* or graduate*, all fields: 2,031,164 results.
3. training or education* or speciali* or "professional development", all fields: 3,005,055 results.
4. #1 and #2 and #3: 6,619 results.
5. (Interdisciplinary or Transdisciplinary or "Multidisciplinary Team" or "MDT" or "Collaborative Work") or (communication or dialogue or conversation or talk or discussion or messaging or exchange) or ("Patient-doctor dialogue" or "Medical communication" or "Patient-centered communication" or "Healthcare communication" or "Doctor-patient interaction" or "Patient-provider communication" or "Medical interview" or "Clinical dialogue"), all fields: 3,396,987 results.
6. #4 and #5: 1372 results.

**Embase**

<1966 to 2023 October 10th

1. 'radiology'/exp OR radiology OR 'medical imaging'/exp OR 'medical imaging' OR 'diagnostic imaging'/exp OR 'diagnostic imaging' OR 'diagnostic radiology':ab,ti,kw; 1,330,258 results.
2. (resident* OR residenc* OR 'clinical attachment*' OR 'clinical rotation*' OR fellowship OR internship OR physician* OR trainee* OR postgraduate* OR graduate*) NEAR/5 (training OR education* OR speciali* OR 'professional development'); 225,257 results.
3. #1 AND #2; 9188 results.
4. interdisciplinary OR transdisciplinary OR 'multidisciplinary team' OR 'mdt' OR 'collaborative work' OR communication OR dialogue OR conversation OR talk OR discussion OR messaging OR exchange OR 'patient-doctor dialogue' OR 'medical communication' OR 'patient-centered communication' OR 'healthcare communication' OR 'doctor-patient interaction' OR 'patient-provider communication' OR 'medical interview' OR 'clinical dialogue'; 2,153,701 results.
5. #3 AND #4; 1,021 results.

**Web of science**

January 1^st^ 1950 to 2023 October 10^th^

1. TS=(radiology OR ‘‘medical imaging’’ OR ‘‘diagnostic imaging’’ OR ‘‘diagnostic radiology’’): 3,480,619 results.
2. TS=((resident* OR fellowship OR "clinical rotation" OR "clinical attachment*" OR fellowship OR internship OR physician* OR trainee* OR postgraduate* OR graduate*) NEAR/5 (training OR education OR speciali* OR "professional development")): 184,833 results.
3. #1 AND #2：8918 results.
4. TS=(Interdisciplinary or Transdisciplinary or "Multidisciplinary Team" or "MDT" or "Collaborative Work"): 225,054 results.
5. TS=(communication or dialogue or conversation or talk or discussion or messaging or exchange): 9,964,716 results.
6. TS=("Healthcare communication" or "Doctor-patient interaction" or "Patient-provider communication" or "Medical interview" or "Clinical dialogue"): 5309 results.
7. #4 OR #5 OR #6: 10,127,696 results.
8. #3 AND #7: 1451 results.
9. Exclude meeting abstracts: 1,166 results.

**ERIC**

January 1^st^ 1950 to 2023 October 26^th^

radiology AND (resident* OR fellowship OR "clinical rotation" OR "clinical attachment*" OR fellowship OR internship OR physician* OR trainee* OR postgraduate* OR graduate*) AND (interdisciplinary OR transdisciplinary OR 'multidisciplinary team' OR 'mdt' OR 'collaborative work' OR communication OR dialogue OR conversation OR talk OR discussion OR messaging OR exchange OR 'patient-doctor dialogue' OR 'medical communication' OR 'patient-centered communication' OR 'healthcare communication' OR 'doctor-patient interaction' OR 'patient-provider communication' OR 'medical interview' OR 'clinical dialogue'): 90 results.
